# Successful Omalizumab Treatment of Rhinogenic Contact Point Headache Complicated by Severe Cedar Pollinosis: A Case Report

**DOI:** 10.7759/cureus.51046

**Published:** 2023-12-24

**Authors:** Isao Suzaki, Naoto Miyoshi, Takahiro Ishima, Kojiro Hirano, Toshikazu Shimane

**Affiliations:** 1 Department of Otorhinolaryngology-Head and Neck Surgery, School of Medicine, Showa University, Tokyo, JPN

**Keywords:** rhinogenic contact point headache, children, omalizumab, pollinosis, seasonal allergic rhinitis

## Abstract

Headache is one of the most common neurological disorders in children. The most common headache in children is a primary headache, including migraine and tension-type headache, but note that secondary headaches should be differentiated as a cause of headache in pediatric patients. The management of cedar pollinosis in pediatric patients is important because it can cause quality-of-life deficits in addition to nasal and ocular symptoms. Omalizumab, an anti-immunoglobulin E (IgE) monoclonal antibody, is approved in Japan as an add-on treatment option for severe cedar pollinosis, but few studies have investigated its real-world clinical efficacy in pediatric patients with seasonal allergic rhinitis. We report the case of a 15-year-old male patient with cedar pollinosis who suffered from uncontrolled naso-ocular symptoms, facial pain, and headache despite using histamine H_1_-receptor antagonists and intranasal corticosteroid spray. A sinus computed tomography scan and nasal endoscopic findings showed a swollen inferior turbinate and nasal septum in contact with the nasal cavity ipsilateral to the headache. Application of local anesthesia to the contact points within the nasal cavity resulted in the rapid relief of headaches. Therefore, we diagnosed rhinogenic contact point headache triggered by cedar pollinosis and initiated the add-on therapy of omalizumab for seasonal allergic rhinitis. Three days after the administration of omalizumab, his naso-ocular symptoms, quality-of-life deficits, and headache improved markedly, accompanied by improved nasal endoscopic findings. Omalizumab was immediately effective for the treatment of rhinogenic contact point headaches complicated by severe cedar pollinosis in a pediatric patient.

## Introduction

Headache is one of the most common symptoms in children, affecting 88% of the child and adolescent population [1]. For children, persistent headaches can cause significant quality-of-life disturbances, including missed schoolwork and extracurricular activities. The most common chronic recurrent headaches in children and adolescents are migraine and tension-type headaches, which are primary headaches [2]. Therefore, the diagnosis and treatment of pediatric patients presenting with headaches should first be based on primary headaches, although it should be noted that headache in children is often associated with secondary headaches.

Allergic rhinitis is one of the most common allergic diseases worldwide, affecting more than 500 million people [3]. Japanese cedar pollen is the most common sensitizing antigen for seasonal allergic rhinitis in Japan, with a prevalence of 38.8%, which is increasing [4]. An Internet survey reported that more than 90% of patients with cedar pollinosis had moderate/severe symptoms based on the Allergic Rhinitis and its Impact on Asthma (ARIA) guidelines symptom scale [5,6]. In addition to nasal and ocular symptoms, such as sneezing, nasal discharge, nasal obstruction, and ocular pruritis, cedar pollinosis reduces patients’ quality of life by causing various neurologic symptoms, including fatigue, cognitive impairment, and mood disorders. The management of allergic rhinitis in children and adolescents is important because allergic rhinitis is associated with sleep disturbances and impaired learning performance. Allergic rhinitis in children rarely resolves spontaneously and usually requires long-term treatment. Standard treatment for seasonal allergic rhinitis is intranasal corticosteroid spray (INCS) and histamine H1-receptor antagonists (antihistamines), but symptoms are difficult to control in severe cases. In Japan, omalizumab, an anti-immunoglobulin E (IgE) monoclonal antibody, is approved as an add-on therapy for seasonal allergic rhinitis in patients aged 12 years and older who have difficulty controlling symptoms despite standard treatment [4]. However, there are few reports of the clinical efficacy of omalizumab in severe allergic rhinitis in children and adolescents in clinical practice.

Rhinogenic contact point headache (RCPH) is defined as headache symptoms associated with contact between the lateral nasal wall mucosa and the nasal septum [7]. According to the third edition of the International Classification of Headache Disorders (ICHD-3), RCPH is classified as a secondary headache or facial pain attributable to a disorder of the nasal mucosa, turbinates, or septum [8]. Abnormal nasal morphology, such as concha bullosa and nasal septal spur, and hyperplasia and inflammation of the mucosa of the nasal cavity are the most common causes of RCPH [7]. Here, we present a case of RCPH associated with severe cedar pollinosis in a child who had a significant response to omalizumab therapy.

## Case presentation

**Figure 1:**
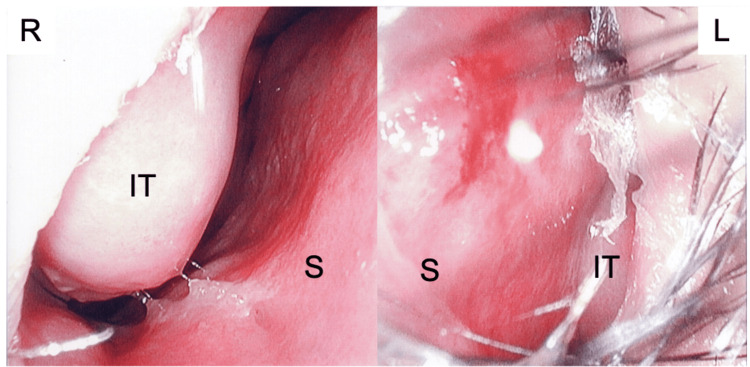
Nasal endoscopic findings at initial examination Nasal endoscopy showed a deviated nasal septum to the left, with mucosal congestion, which was in contact with a swollen inferior turbinate. IT: inferior turbinate, S: nasal septum, R: right, L: left

A 15-year-old boy was referred to our hospital because he had uncontrolled sneezing, nasal discharge, blockage, itching, left-side headache, and facial pain despite using an antihistamine, a leukotriene receptor antagonist, and INCS therapy. The patient had had seasonal and perennial allergic rhinitis since childhood, with nasal and ocular symptoms throughout the year, and his symptoms worsened annually during the cedar pollen dispersal period. From the age of 13 years, the patient had headaches and facial pain on the left side, in addition to nasal and ocular symptoms, during the cedar pollen dispersal period. A neurologic examination, including a brain magnetic resonance imaging scan, had been previously performed, but no obvious abnormal neurologic findings were noted. Treatments for migraine headaches had been offered in other clinics, but their therapeutic effects were inadequate. We performed a blood test, which showed an elevated total IgE level (959 IU/mL). The ImmunoCAP (Thermo Fisher Scientific, Waltham, MA, USA) specific IgE blood test was positive for multiple seasonal and perennial antigens including Japanese cedar pollen (>100.0 UA/mL, class 6), cypress pollen (62.5 UA/mL, class 5), ragweed pollen (54.0 UA/mL, class 5), and house dust mite (12.2 UA/mL, class 3). Nasal endoscopy showed a nasal septum deviated to the left with mucosal congestion. The nasal septum was in contact with a swollen inferior nasal turbinate. We could not observe the posterior part of the left nasal cavity due to the narrowing of the nasal cavity (Figure 1).

**Figure 2:**
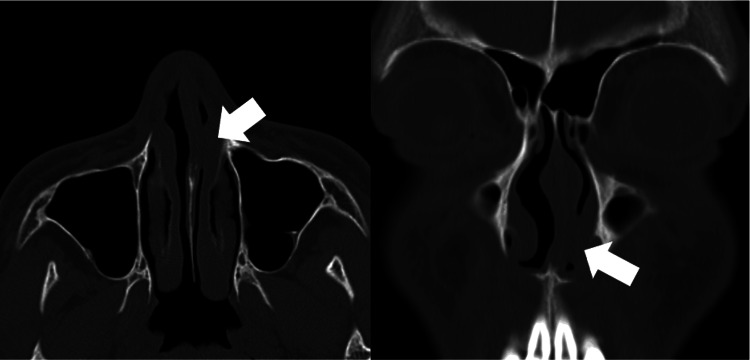
Sinus computed tomography images Sinus computed tomography showed contact between the nasal septum and the left inferior turbinate (white arrow).

A sinus computed tomography scan showed contact between the nasal septum and the left inferior turbinate but no rhinosinusitis (Figure 2).

Application of local anesthesia to the contact points within the left nasal cavity resulted in the rapid relief of headache and facial pain.

Based on the patient’s history and clinical findings, we diagnosed severe cedar pollinosis accompanied by RCPH. Omalizumab 600 mg for severe seasonal allergic rhinitis was initiated as an additional treatment to INCS and antihistamines to enhance the treatment of allergic inflammatory control of the nasal mucosa. We evaluated the patient’s symptoms of allergic rhinitis using the Japanese Rhinoconjunctivitis Quality of Life Questionnaire (JRQLQ) (0: none, 4: worst) [4]. We evaluated headache symptoms using a visual analog scale (0: none, 10: worst). The clinical course before and after omalizumab treatment is shown in Table 1.

**Table 1:** Clinical course before and after omalizumab treatment The symptoms of allergic rhinitis were evaluated using the Japanese Rhinoconjunctivitis Quality of Life Questionnaire (JRQLQ) (0: none, 4: worst). The symptoms of headache were evaluated using a visual analog scale (0: none, 10: worst). All symptoms, including headache, improved markedly after 2 weeks of omalizumab treatment.

	Before omalizumab administration	Two weeks after omalizumab administration
Self-reported Japanese Rhinoconjunctivitis Quality of Life Questionnaire (JRQLQ)		
Nasal and eye symptoms		
Runny nose	4	1
Sneezing	2	1
Blocked nose	3	0
Itchy nose	4	1
Itchy eyes	2	1
Watery eyes	1	0
Rhinitis-related QOL scores		
Usual daily activities	1.2	0
Outdoor activities	2.5	0
Social functioning	0.7	0
Sleep problems	2	0
General physical function	1	0
Emotional function	0.5	0
Global status		
Face scale	2	0
Visual analog scale for symptoms		
Headache	7.6	0

**Figure 3:**
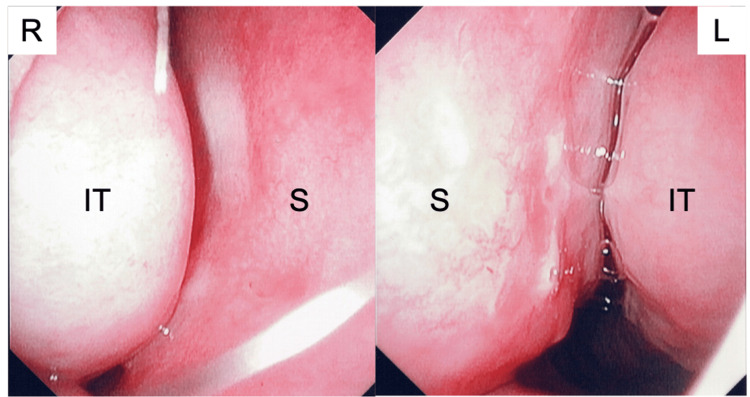
Nasal endoscopic findings two weeks after the initiation of omalizumab administration. Nasal endoscopy showed that the swelling and erosions of the nasal mucosa had improved. IT: inferior turbinate, S: nasal septum, R: right, L: left.

All symptoms, including nasal and ocular symptoms and headache, improved markedly from three days after the induction of omalizumab treatment. Moreover, two weeks after the initiation of omalizumab, an improvement in nasal mucosal swelling and congestion of the nasal septal mucosa was observed by nasal endoscopy (Figure 3).

Only one dose of omalizumab was administered in early March during the cedar pollen dispersal period. Throughout the cedar pollen dispersal season, the patient was able to control his symptoms, including nasal, eye, and headache symptoms, without additional rescue medication such as oral corticosteroids (OCS), nasal decongestants, and analgesics. When the cedar pollen dispersal ended, the patient did not experience a headache again after discontinuing omalizumab.

## Discussion

Omalizumab specifically binds to free IgE and inhibits allergic reactions by blocking IgE binding to the surface of mast cells and basophils. Omalizumab is indicated for the treatment of moderate-to-severe allergic asthma and chronic spontaneous/idiopathic urticaria worldwide because it prevents the activation of the IgE-mediated allergic cascade [9]. Furthermore, in patients with inadequately controlled cedar pollinosis despite combined INCS and antihistamine therapy, the add-on administration of omalizumab significantly improved nasal and ocular symptoms during the peak cedar pollen dispersal period compared with placebo [10]. Omalizumab treatment of patients with severe cedar pollinosis also reduced the loss of labor productivity by approximately one-third [11]. Based on the results of a phase 3 study, omalizumab was approved in December 2019 in Japan for use in patients with inadequately controlled severe seasonal allergic rhinitis, despite standard treatment [4,10]. The authors reported the results of a prospective observational study of 18 patients with cedar pollinosis where add-on treatment with omalizumab at the peak of cedar pollen dispersal was effective at rapidly relieving symptoms [12]. Although omalizumab may be more effective if initiated before symptoms are exacerbated at the peak of pollen dispersal, in this case report, the patient showed a significant response to omalizumab, even after the development of symptoms, including headache.

In this case, all diagnostic criteria for headache secondary to a disorder of the nasal mucosa, turbinate, or nasal septum were demonstrated: the development of a headache led to the discovery of a nasal mucosal contact point; the headache significantly improved in parallel with improvement of the nasal lesion after add-on treatment for allergic rhinitis; the headache significantly improved following local anesthesia of the mucosa in the region of the lesion; and the headache developed ipsilateral to the site of contact between the nasal septum and the inferior turbinate [8]. The fact that the onset of headache in this patient coincided with the cedar pollen dispersal period every year when nasal mucosal swelling worsens also supports this diagnosis. Migraine is the most common cause of chronic recurrent headaches in pediatric patients, and the diagnosis and treatment of primary headaches are fundamental. Although there are limited literature reports of RCPH cases in children, the possibility of secondary headaches, including RCPH, should be considered in pediatric cases of refractory headaches despite standard treatment for migraine [13,14].

The mechanism of RCPH is postulated to be that mechanical stress from contact between the nasal septum and the nasal mucosa induces substance P release from the nasal mucosa, which in turn stimulates sensory C-fibers. Substance P was also reported to induce local responses, such as vasodilation, plasma extravasation, and mucus hypersecretion, further intensifying nasal contact sites and exacerbating headaches [15,16]. The first-line treatment of RCPH includes conservative treatment with INCS and antihistamines to reduce inflammation of the nasal mucosa and alleviate nasal mucosal contact, whereas, for refractory cases, surgical removal of the in-contact nasal mucosa is a treatment option [7]. In this case, the timing of the RCPH coincided with the cedar pollen dispersal period, when seasonal allergic rhinitis worsens, so it was appropriate to first try supplemental anti-inflammatory treatment before considering surgical treatment. A possible mechanism by which omalizumab was effective in RCPH associated with pollinosis was the improvement in the edema of the nasal mucosa as demonstrated by nasal endoscopic findings (Figures 1, 3). For cases of allergic rhinitis with poor response to the combination therapy of antihistamines and INCS, it is important to review basic treatment principles, including avoiding antigen exposure and checking medication compliance. If symptoms are poorly controlled despite good compliance and avoidance of antigen exposure, the addition of a short-term OCS and/or nasal decongestant could be a treatment option. However, frequent or prolonged OCS administration should be avoided because of associated side effects, especially in children and adolescents. Moreover, although nasal decongestants may be effective for the temporary relief of symptoms such as nasal congestion, the long-term and habitual use of a nasal decongestant may induce rhinitis medicamentosa. Allergen immunotherapy is a curative treatment for allergic rhinitis and should be considered as a long-term treatment option, but it is not expected to provide immediate symptomatic relief. There have been several reports showing that surgical treatment for RCPH in children reduced the severity of symptoms [17-19]. However, the effectiveness of surgical intervention for RCPH is controversial. In particular, nasal morphology-improving surgery, including septoplasty and turbinectomy, in children requires more careful judgment than in adults when deciding on the indications for surgery due to concerns about the impact on the development of maxillofacial morphology. Therefore, we think that the appropriate control of inflammation by conservative treatment should be attempted first for pediatric patients with RCPH with allergic rhinitis. Omalizumab therapy for refractory allergic diseases, including severe seasonal allergic rhinitis with various disease burdens, such as RCPH, is an effective treatment option with immediate results and few side effects. To the best of our knowledge, this is the first report of successful omalizumab treatment in a patient with RCPH with severe seasonal allergic rhinitis.

## Conclusions

Seasonal allergic rhinitis in pediatric patients may have a varied symptom burden, including headache, in addition to nasal and ocular symptoms. Secondary headaches should be considered in pediatric cases of refractory headaches despite standard treatment for migraine. Omalizumab may be an effective treatment option for a variety of symptoms associated with refractory seasonal allergic rhinitis.
